# Computer vision-based instantaneous speed tracking system for measuring the subtask speed in the 100-meter sprinter: Development and concurrent validity study

**DOI:** 10.1016/j.heliyon.2024.e24086

**Published:** 2024-01-06

**Authors:** Teerawat Kamnardsiri, Sirinun Boripuntakul, Chinawat Kaiket

**Affiliations:** a Department of Digital Game, College of Arts, Media and Technology, Chiang Mai University, 50200, Chiang Mai, Thailand; b Department of Physical Therapy, Faculty of Associated Medical Sciences, Chiang Mai University, 50200, Chiang Mai, Thailand; c Department of Vocational Education and Wellness Promotion, Faculty of Education, Chiang Mai University, 50200, Chiang Mai, Thailand; d Department of Sport Sciences, Graduate School, Chiang Mai University, 50200, Chiang Mai, Thailand

**Keywords:** Speed detection, Computer vision, Human detection, Sprinter, Athletics, Image processing, Video analysis, Speed test, 100-M sprint

## Abstract

The 100-m sprint is one of the track events, and the pace of the runner can be measured using a variety of tools, such as a hand stopwatch, timing gate, laser device, radar device, photocell timing, etc. The data measured is the mean travel time. Nonetheless, monitoring an individual sprinter's instantaneous speed tracking is essential for assisting staff trainers in developing an appropriate training schedule for the individual sprinter. The purpose of this study was to construct a computer vision-based system for assessing the sprinting speed of the 100-m subtasks and also to investigate the concurrent validity of video analysis software among athletes. Five athletes participated in the research to determine its validity. Over the course of two trials, the sprinting pace of each participant's subtasks (a 100-m split to 10 m for each subtask) was measured. The application of the computer vision-based system to video analysis software was validated using the Pearson correlation coefficient. The agreement between the two measurement systems was quantified using Bland-Altman plots. The results revealed a significant relationship between the two systems and all 100-m subtask sprinting speeds (r = 0.961–1.000, p 0.0001). The Bland-Altman analyses indicated that the mean differences in 100-m subtask speeds were consistently close to zero, falling within the 95 % limits of agreement. The scatter plot distribution showed symmetry. The computer vision-based system proved to be a valid tool, suggesting its potential value in measuring and monitoring the 100-m subtask sprinting speed of athletes.

## 1 Introduction

Athletics is one of the most prevalent exercise options [[1], [2], [3], [4]]. An estimated one hundred million individuals are enthusiastic spectators and participants of athletics, with the sport enjoying regional prominence in the Americas, Asia, Africa, Europe, Oceania, North and Central America, the Caribbean, and South America [2]. The Edudwar website's report on the 15 most popular sports worldwide in 2023 revealed that athletics is adored by an audience of around 300 million people [3]. There are two different categories of events in Track and Field. Track events involve short-running, medium-running, and long-distance running, whereas field events consist of jumping and throwing [5]. The 100-m sprint is a track event that can be categorized into three primary phases: (1) the phase of acceleration, (2) the phase of maximum velocity, and (3) the phase of deceleration [[6], [7], [8], [9]]. Moreover, sprint running involves a cyclical motion; the supporting phase and the non-supporting phase are the two primary phases of the cyclic running movement [10]. If sprinter needs to make good speed, they have to run as fast as possible. Therefore, the performances of the sprinters are enhanced by utilizing essential sprint metrics, including split times, split distance, step length, step length, and ground contact time. Previous research and coaching practices have utilized many sprint characteristics in order to enhance sprint performance in both training and competitive settings [[11], [12], [13], [14], [15], [16]].

Besides, numerous specific training strategies, such as biomechanics, strength, speed, and endurance, are considered for enhancing the performance of athletes. Furthermore, sports science and technology have an effect on athletes, such as sensor monitoring technology, computer vision techniques, high-speed video cameras, digital image and signal processing, etc. There are still challenges and numerous research opportunities for enhancing the athletic performance of each individual.

The biggest problem with staff coaches is that general coaches are unable to determine immediate and maximum speed during athletes’ running using only their eyes and time from a hand stopwatch. The staff coach only understands how to catch average speed time from a hand stopwatch, which has more mistakes than the gold standard system. As a result, instructors may be unable to recommend and construct practice plans for particular athletes. Furthermore, it could be the secret to increasing athletes' performance.

This study makes significant contributions to the existing literature in a crucial field, emphasizing the development of a computer vision-based system for instantly tracking the speed of an individual sprint athlete. The objective is to establish a computer vision system specifically designed for monitoring the immediate speed of 100-m sprinters. Ultimately, the system's performance needs to be validated against an already established tool.

## 2 Literature review

Nowadays, the development of digital equipment supports the use of technology to produce automatic applications. In computer science research, computer vision techniques were used to analyze the human movement.

Fast speed detection, the speed of the human running can be measured with several tools, such as a timing gate [17], hand stopwatch [18], laser device [[19], [20], [21], [22]], radar device [23], motion capture system [[24], [25], [26], [27], [28]], photocell timing [25,29], video timing [24,26,[30], [31], [32], [33], [34], [35], [36], [37]], Global Positioning Systems (GPS) [17,38,39], Global Navigation Satellite System (GNSS) [40], radio-based tracking system [41], and Inertial Measurement Unit (IMU) [[42], [43], [44]].

The action recognition system aims to automatically analyze the human movements made by a human agent during the performance of a task into actions. The typical action recognition system consists of feature extraction, action learning, action classification, and action segmentation [45]. For instance, Eveland et al. [46] employed background modeling for the segmentation of video-rate stereo sequences. And also, Kamnardsiri et al. [26] developed a speed detection system for measuring the instantaneous walking speed of older adults. Nagano et al. [24] studied a new low-cost and time-efficient methodology for determining instantaneous horizontal walking and running speed. Kamnardsiri [33] designed a system to detect speed, measuring the immediate running speed of seven sprinters. The setup involved a video camera recording a 25-m running distance. The findings revealed a highly significant correlation between the two systems, with Spearman's Rho Correlation exceeding 0.99 at a p-value below 0.0001.

The previous study conducted by the video analysis software (Dartfish) [30] provided evidence that the latter measuring approach was valid within the constraints of its accuracy. The instruments have a precision of around ±0.01 s. The video data were captured by a video camera (Sony DCRPC105E) and then calculated velocities that ted a near-perfect correlation (r = 0.99) after analyzing the data [31]. The time was compared with photocells [32], which documented the use of an Intraclass Correlation Coefficient (ICC). When the velocity data were evaluated using a statistical analysis, a correlation coefficient of 0.98 was obtained. Cameras with frame rates of 50 Hz and 100 Hz.

Moreover, Kamnardsiri et al. [47] developed a Knowledge-Based Smart Trainer (KBST) system for coaching in long jump, utilizing computer vision and image processing methods. They examined the effectiveness of the system in assessing the performance of long jump students and evaluated the transfer of knowledge from coaches to students. The study utilized two video cameras to record the speed and jump angle of twenty-two long jumpers. The KBST system proved to be successful in enhancing the performance of long jump students and facilitating the transfer of knowledge from coaches to the students.

The study designed the approach [48] that produced the number of steps, stride length, and speed transition from a film of a 100-m sprint using OpenPose. The results demonstrated the method's correctness by comparing it to data measured using standard methods. Furthermore, the accuracy of estimating the number of steps and displaying visualized runners' steps and speed transitions. Stenum et al. [49] compared spatiotemporal and sagittal kinematic gait parameters derived from OpenPose with those obtained using 3D motion capture during overground walking in healthy adults. The results presented that the described method effectively and accurately computes spatiotemporal gait parameters, as well as hip and knee angles. This capacity is well-suited for detecting alterations in the gait pattern. Yang et al. [50] employed OpenCV to recognize and track middle-distance runners in a 1500 m race video, determining their actual coordinates, running distance, and speed. Using a 4 K high-definition camera and AutoCAD for playground mapping, OpenCV is utilized for image recognition and tracking. Results show discrepancies in the runner's actual distance covered compared to the official distance, but speed calculations using object tracking align closely with manual video analysis. Despite challenges, this pioneering use of object tracking in 1500 m middle-distance running offers valuable insights for future race analysis.

García-Pinillos et al. [51] evaluated the precision of two wearable devices (Stryd™ and RunScribe™) in gauging spatiotemporal parameters while running on a treadmill, as compared to high-speed video analysis conducted at a rate of 1000 Hz. The study involved forty-nine endurance runners, and the parameters under scrutiny included step length, step frequency, contact time, and flight time. The results indicated that both foot pods were considered reliable for measuring spatiotemporal parameters during treadmill running at a comfortable pace. Notably, the RunScribe™ system demonstrated greater accuracy in temporal parameters and SL compared to the Stryd™ system, taking into account the agreement limits with high-speed VA.

Jafarzadeh et al. [52] introduced a real-time 2D athlete pose estimation system using OpenPose, capturing body part positions in a hurdles athlete, and tested it with videos from a top-tier hurdler. The study assessed the performance of various feature extractors in OpenPose, revealing that the system with the extended version of VGG19 as the feature extractor demonstrated superior accuracy compared to other algorithms.

Felipe et al. [53] demonstrated the concordance between a multi-camera tracking system (Mediacoach®) and a GPS system for real-time monitoring of movements in elite football players. The findings indicated strong agreement with high Intraclass Correlation Coefficient (ICC) values (>0.75) and substantial correlations (r > 0.70) across all variables. However, the video-based system exhibited a tendency to overestimate results in various speed zones, number of sprints, and maximum speed. The study concluded that, particularly for total distance and distances covered at medium speeds, Mediacoach® is comparable in accuracy to a GPS system, providing real-time, objective data tailored to the physical and movement requirements of elite football.

The literature review highlights the potential application of computer vision and image processing techniques in monitoring the immediate speed of individual sprint athletes, aiding coaches in devising tailored training plans. Thus, the study focuses on developing a computer vision-based system for tracking the instantaneous speed of 100-m sprinters. The primary objective is to evaluate the simultaneous accuracy of this system in measuring the speed of each subtask within the 100-m sprint. Additionally, the study evaluates the concurrent validity of each subtask's sprint performance using video analysis software, Tracker 6.1.3 [54], as the analysis and modeling tool.

## 3 Materials and methods

### 3.1 Study design

This validity study was a prospective 100-m speed trial, a cross-sectional study design, with two trials of 100-m sprinting to evaluate the concurrent validity between the computer vision-based system and video analysis software. The research procedure received approval from the Human Ethical Review Board at the institution of the principal investigator (approval number: AMSEC-65EX-017). Prior to enrollment, all participants provided written consent after receiving relevant information.

### 3.2 Recruitment and participants

Five sprint athletes were recruited from Chiang Mai University, Chiang Mai, Thailand. Participants performed sprinting 100-m as fast as possible two times to evaluate sprinting performance. The inclusion criteria were: (1) Thai national healthy sprint athletes age 18–25 years; (2) able to do running fast as 4 m s^−1^ or more 100 m; and (3) able to comprehend instructions and willing to participate. Exclusion criteria were having: (1) The presence of physical conditions such as a musculoskeletal injury that preclude participants from completing the testing protocol; (2) Taking alcohol 6 h before testing or using drug regimens that affect running performance.

### 3.3 Equipment and experimental setup of the proposed system

**Fig. 1:**
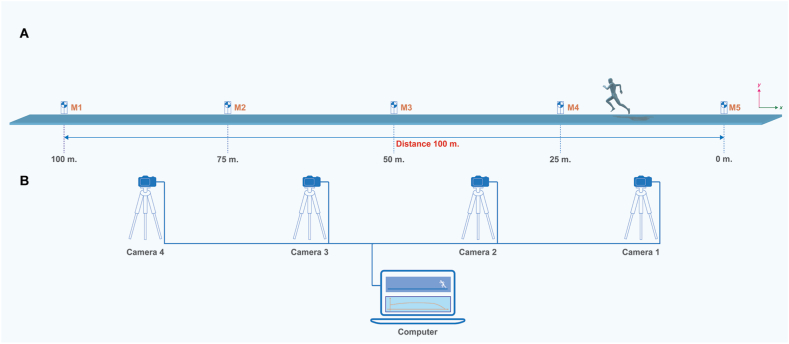
Experimental setup of the 100-m sprint test (A) Sprint test of the participant and the position of markers (M1 to M5), and (B) The position of web cameras (Camera 1 to Camera 4) and the computer with installed the MultiCam capture software.

The subtask speed of the 100-m speed (m.s^−1^) was separated into 10 m for each subtask. It was evaluated using the computer vision-based system with four standard web cameras (Logitech C920 HD Pro Webcam, Logitech®, China). The computer vision-based system generated positional data for each participant as a 2D coordinate system (X-axis and Y-axis). Each video camera was set at 30 Hz. The resolution of each video was 640 × 360 pixels. Four video clips of each speed test were collected using the multi-camera video capture software (MultiCam Capture 2.0, Corel® Corporation, Canada). Furthermore, the development of the computer vision-based system involved utilizing MATLAB® 2015a from MathWorks, Inc., Natick, Massachusetts, USA. Moreover, the Computer Vision and Image Processing Toolbox were employed to detect the center of the human body. The collected data from all sources was combined with the interpolate missing values function (fillmiss.m, Kirill K. Pankratov, MIT, USA) and then filtered with a smooth function. The markers were placed at intervals of 100 m, starting from (M5) and extending to the end of the 100-m running track (M1), as depicted in Fig. 1A. Furthermore, each webcam was positioned at a height of 1.2 m above the ground and located approximately 20 m away from the running track used for testing. Data collection was conducted at the main stadium of Chiang Mai University, Chiang Mai, Thailand. The experimental configuration is illustrated in Fig. 1B.

### 3.4 Assessment of the concurrent validity of the proposed system

Participant preparation: The eligible participants were informed about the study's purposes before signing informed consent. And then, all participants warm up for the body, including stretching, dynamic exercises, and jogging, to prepare their muscles around 30–45 min before the test. Additionally, before participating in the 100-m activity, participants were instructed to don a black, long-sleeved sports shirt and black athletic jogger pants. After that, they performed the sprint on the synthetic running track, flat and dry. All participants performed the sprint test on two attempts. After the first attempt, they were asked to take a rest for around 15 min and then test the second attempt, respectively.

### 3.5 Computer vision-based for processing the 100-m speed test

**Fig. 2:**
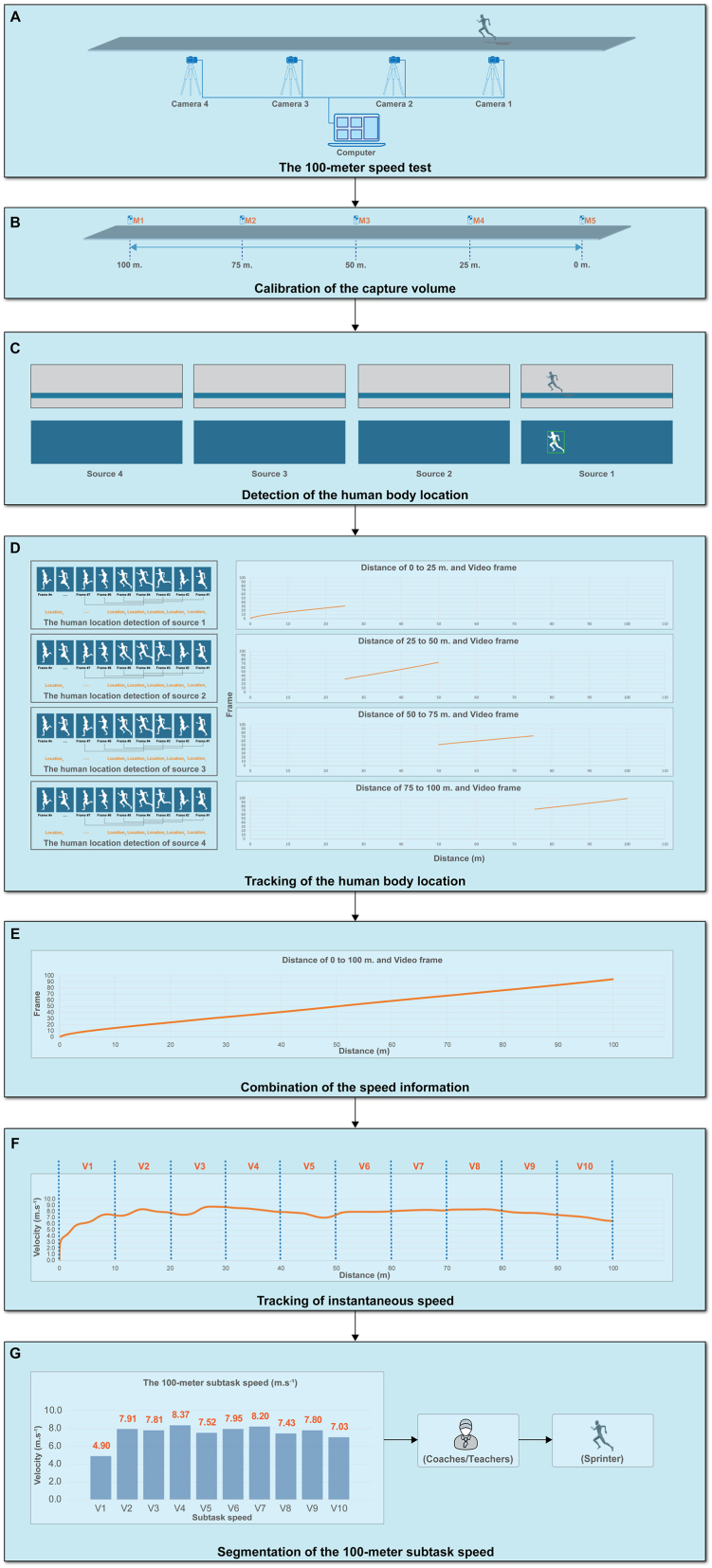
The diagram of the processing in the proposed system for detecting the speed of the 100-m subtask (A) The 100-m speed test of each participant, (B) Calibration of the capture volume with positions of M1 to M5 markers, (C) Detection of the human body location of each Source 1 to Source 4, (D) Tracking of the body location of four sources, (E) Combination of the speed information into the 100-m distance data, (F) Tracking of the instantaneous speed of the participant, and (G) Segmentation of the 100-m subtask speed of each 10 m.

The processing steps for calculating the speeds of the 100-m subtasks in our computer vision-based system were segmented into seven stages, as illustrated in Fig. 2.

#### 3.5.1 The 100-m speed test

In this stage, the participant prepared to begin at the starting point. The contestant received the starting signal (whistle sound) from the starting referee. The participant completed the 100-m run as quickly as possible to the endpoint. During the run, all video recordings were collected by four conventional web cameras using the Corel® MultiCam Capture 2.0 software that was loaded on the laptop. The starting and ending points of all video files were synchronized, as shown in Fig. 2A.

#### 3.5.2 Calibration of the capture volume

The marker was placed every 25 m from the starting point (0-m) to the end of the running test point (100-m) to calibrate the proposed system. Fig. 2B shows how these markers were used to determine the capture volume of the 100-m in the horizontal plane (x-axis).

#### 3.5.3 Detection of the human body location

In this step, the human body (participant) location was detected by employing the background subtraction technique (motion segmentation and feature extraction) within the video frames of each video file that presents in Fig. 2C. The foreground detection $\left(F_{d}\right)$ as given in equation (1):

(1) $$F_{d}\left(x,y\right)=F\left(x,y\right)-B\left(x,y\right)$$

where F(x,y) represents the foreground pixel value, B(x,y) represents the background pixel value that was captured before testing, and $F_{d}\left(x,y\right)$ represents the foreground detection pixel value in each video frame. The foreground and background were set as grayscale values (0–255).

To remove the background from each image, use the Thresholding technique and then convert to a grayscale image as given in equations (2), (3), (4), (5):

(2) $$H_{Body}\left(x,y\right)=\left\{ \begin{array}{c} 0\;if\;F_{d}\left(x,y\right)>\;\tau \\ 255\;if\;F_{d}\left(x,y\right)\leq \tau \end{array}\right.$$

where $H_{Body}\left(x,y\right)$ represents the human body pixel value, τ is a Threshold value. In this study the Threshold was set as τ=40 in any initial experimental results. This threshold value was used for all video frames in this study.

To remove noise, a 2D median filtering (3 × 3 adjacent pixels) was used to eliminate noises from $H_{Body}$ of each image. Subsequently, the resulting image was converted to a binary representation, where black is denoted by *‘0′* and white is represented by *‘1′* as outlined in equation (3):

(3) $$H_{Binary}\left(x,y\right)=\left\{ \begin{array}{c} 0\;if\;H_{body}\left(x,y\right)=0\\ 1\;if\;H_{body}\left(x,y\right)>0 \end{array}\right.$$

where $H_{Binary}$ represents the human body binary image.

The Bounding Box of a BLOB Analysis [55] was used to locate the geometric center of mass (COM) location. The centroid of each participant was marked to identify their position. $H_{COM}\left(x_{centroid},y_{centroid}\right)$, $x_{centroid}$ and $y_{centroid}$ of N pixels placed at location $x:\left(x_{i}\right)$ and position $y:\left(y_{i}\right)$ as given in equations (4), (5):

(4) $$x_{centroid}=\frac{1}{N}\sum_{i=1}^{N}x_{i}$$

(5) $$y_{centroid}=\frac{1}{N}\sum_{i=1}^{N}y_{i}$$

where N represents the number of pixels in the $H_{Binary}$ detected by a BLOB Analysis, and $\left(x_{i},y_{i}\right)$ is the x and y coordinates of the pixels, respectively.

#### 3.5.4 Tracking of the human body location

To track the human body centroid position, real centroid of the human body in pixel value (${Body}_{pixel}$) was considered to calculate and also calibrated values of the 100-m position (M1), the 75-m position (M2), the 50-m position (M3), the 25-m position (M4), and the 0-m position (M5) were performed for tracking the real position of the human body COM location ($H_{RealLoc}$) as given in equations (6), (7), (8), (9):

(6) $$H_{RealLoc}={Body}_{pixel}\times {OnePixel}_{distance}$$

(7) $${Body}_{pixel}=x_{centroid\;}-\;M_{\text{end}}$$

(8) $${OnePixel}_{distance}=\frac{25}{{Cal}_{volume}}$$

(9) $${Cal}_{volume}=M_{\text{start}}-\;M_{\text{end}}$$

where, ${OnePixel}_{distance}$ represents the value of distance of each pixel, $x_{centroid}$ represents the human body centroid position, ${Cal}_{volume}$ represents the distance value between $M_{\text{start}}$ and $M_{\text{end}}$ , $M_{\text{start}}$ represents the start point of the video image, $M_{\text{end}}$ represents the end point of the video image, and 25 is the actual distance between the start point marker (M1) and the endpoint marker (M2) (for instance, the distance between M1 and M2 is comparable to 25 m of video from Source 4).

Following that, data from videos Source 1 (markers M4 and M5), Source 2 (markers M3 and M4), Source 3 (markers M2 and M3), and Source 4 (markers M1 and M2) were calculated to track the actual position of the human body COM location that presents in Fig. 2D.

#### 3.5.5 Combination of the speed information

Each period of data from the video source was calculated independently for tracking the human body's COM location. As a result, in order to integrate the human body location, these data were combined into a single data set (distance 0 m–100 m). Fig. 2E illustrates the combination of the human body COM location data ($H_{Combine}$) as given in equation (10):

(10) $$H_{Combine}\left(loc\right)=H_{1}+H_{2}+H_{3}+H_{4}$$

where,

$$H_{1}=m_{1}loc+b_{1};\;0<loc\leq 25\text{,}$$

$$H_{2}=m_{2}loc+b_{2};\;25<loc\leq 50\text{,}$$

$$H_{3}=m_{3}loc+b_{3\;};\;50<loc\leq 75\text{,}$$

$$H_{4}=m_{4}loc+b_{4};\;75<loc\leq 100\text{,}$$

$$m_{1}=\frac{{\Delta F}_{1}}{{\Delta loc}_{1}}\text{,}$$

$$m_{2}=\frac{{\Delta F}_{2}}{{\Delta loc}_{2}}\text{,}$$

$$m_{3}=\frac{{\Delta F}_{3}}{{\Delta loc}_{3}}\text{,}$$

$$m_{4}=\frac{{\Delta F}_{4}}{{\Delta loc}_{4}}$$

and, loc represents the human body's centroid location, F represents the position of frame of the video, $b_{1},b_{2},b_{3,}\;b_{4}$ represent a constant of each distance, ${m_{1},m}_{2},m_{3},m_{4},m_{5}$ represent the difference of frame ${\Delta F}_{1}$, ${\Delta F}_{2}$ , ${\Delta F}_{3}$ , ${\Delta F}_{4}$ divide by the difference of location ${\Delta loc}_{1}$ , ${\Delta loc}_{2}$ , ${\Delta loc}_{3}$ , ${\Delta loc}_{4}$ of each distance, respectively. And also, when a missing value appears at the frame a NaN value will be added in the $H_{Combine}\left(loc\right)$. The tracking location process had some missing values. As a result, a data cleaning process was carried out in order to recover missing values from the human body COM position data ($H_{Combine}$). For recovering missing values, the interpolates missing values function (fillmiss.m, Kirill K. Pankratov, MIT, USA) that presented in Data Analysis: Filtering, Cross-correlation, Coherence and Applications to Geophysical Data using Matlab [56] and then filtered with a smooth function were employed, as shown in Fig. 2E.

#### 3.5.6 Tracking of the instantaneous speed

The human body's COM location in the coordinate system was collected as an array in order to track the velocity of the human. The horizontal plane velocity (${Velocitx}_{Index}$) was calculated for each of the four frames (DurationTime=4) in the video image, following the equations provided in equation (11), (12), (13):

(11) $${Velocitx}_{Index}=\frac{{\Delta x}_{index}}{{\Delta tx}_{index}}$$

(12) $${\Delta x}_{index}={distance}_{index+DurationTime}-\;{distance}_{index}$$

(13) $${\Delta tx}_{index}={time}_{index+DurationTime}-\;{time}_{index}$$

where, ${\Delta x}_{index}$ represents the difference between current distance (${distance}_{index}$) and the distance at the next 4 frames (${distance}_{index+DurationTime}$), and ${\Delta tx}_{index}$ represents the difference between current (${time}_{index}$) time and time at the next 4 frames (${time}_{index+DurationTime}$).

Fig. 2F illustrates that the 100-m subtask velocity segmentation was split into 10 subtasks, including.

a. **Sprinting meter 0 to 10 (V1)** was computed using the horizontal running distance of the human body and the quantity of frames within the range from meter 0 to meter 10.
b. **Sprinting meter 10 to 20 (V2)** was computed using the horizontal running distance of the human body and the quantity of frames within the range from meter 10 to meter 20.
c. **Sprinting meter 20 to 30 (V3)** was computed using the horizontal running distance of the human body and the quantity of frames within the range from meter 20 to meter 30.
d. **Sprinting meter 30 to 40 (V4)** was computed using the horizontal running distance of the human body and the quantity of frames within the range from meter 30 to meter 40.
e. **Sprinting meter 40 to 50 (V5)** was computed using the horizontal running distance of the human body and the quantity of frames within the range from meter 40 to meter 50.
f. **Sprinting meter 50 to 60 (V6)** was computed using the horizontal running distance of the human body and the quantity of frames within the range from meter 50 to meter 60.
g. **Sprinting meter 60 to 70 (V7)** was computed using the horizontal running distance of the human body and the quantity of frames within the range from meter 60 to meter 70.
h. **Sprinting meter 70 to 80 (V8)** was computed using the horizontal running distance of the human body and the quantity of frames within the range from meter 70 to meter 80.
i. **Sprinting meter 80 to 90 (V9)** was computed using the horizontal running distance of the human body and the quantity of frames within the range from meter 80 to meter 90.
j. **Sprinting meter 90 to 100 (V10)** was computed using the horizontal running distance of the human body and the quantity of frames within the range from meter 90 to meter 100.
7. Segmentation of the 100-m subtask speed:

To report the performance test, the mean sprint velocity was split every 10 m to display the participant's performance [10,57,58]. The average velocity data from V1 to V10 was used to compute the velocity values from V1 to V10. Finally, the bar graph was depicted the 100-m subtask speed test as given in equation (14), (15):

(14) $$V=\frac{1}{M}\sum_{n=1}^{NV}Velocity\left(n\right)$$

(15) $$NV=\left\{ \begin{array}{c} \sum_{i=1}^{SV}nv=nv+1,if\left(H\left(i\right)\geq {sp}_{loc}\right)\cap \left(H\left(i\right)\leq {ep}_{loc}\right)\\ end,otherwise.\; \end{array}\right.$$

where, V represents the average of velocity of each period, NV represents the number of Velocity values of each period, SV represents the size of the Velocity array, H(i) represents the location of the human body, ${sp}_{loc}$ is the starting point of each period, and ${ep}_{loc}$ is the endpoint of each period Fig. 2G.

The speed of tracking for each participant and the segmentation of the 100-m subtasks were computed using a MATLAB® 2015a script, incorporating the computer vision and image processing toolbox (The MathWorks, Inc., Natick, Massachusetts, USA). The data capture and computation were carried out on a laptop computer from ASUSTek® Computer Inc., Taipei, Taiwan with Intel® Core (TM) i5-8265U 8th Gen CPU@1.60 GHz, 2 GB NVIDIA graphic card 2 GB RAM, and 8 GB DDR4 RAM.

### 3.6 Video analysis software for processing the 100-m speed test

The Video Analysis and Modeling Tool (Tracker), which is built on the open source physics software [59], is employed to track moving objects and provide their position and velocity. Tracker provides powerful analysis tools that make tracking the human velocity from a video source simple. Several prior research used Tracker to analyze the movement for running the 100-m [60] and to detect the speed of the 25-m sprinter [33]. Therefore, in this study, the expert performed Tracker 6.1.3 as a ground truth of the speed information. Tracker's processing of the 100-m speed test was divided into five parts, as follows.

#### 3.6.1 Loading video data

Each participant's captured video was downloaded onto the laptop. Furthermore, the starting frame, finish frame, and step size (4 frames) were all specified to compute the speed of each video source.

#### 3.6.2 Calibration

Each collected video's capture volume was calibrated using the calibration stick. For example, the starting point (M5) and endpoint (M4) of the calibration stick were marked, and then 25 m were entered into the calibration stick label. Furthermore, the coordinate axes were placed at the starting position to establish the axes' origins as (x=0)and(y=0).

#### 3.6.3 Setup the point mass

In this step, the centroid of the human body was tracked using the point mass from the beginning to the end of each of the four frames.

#### 3.6.4 Calculating and exporting the velocity

The values from the point mass were calculated and then displayed in the Tracker software's table. The velocity information for each was then exported into a. txt file format.

#### 3.6.5 Segmentation of the 100-m subtask speed

Finally, all of the txt files were imported into Microsoft® Excel 2016 Spreadsheet software for the combination of all sources of each participant, and then the video data (100-m speed test) was separated into V1 to V10, accordingly.

### 3.7 Statistical analysis

The concurrent validity of the proposed system in detecting velocities for the 100-m subtasks was evaluated through the Pearson correlation coefficient (r), with a specified probability level of 0.05 to determine statistical significance when compared to the Tracker. The assessment of the relative agreement between the systems was explained as follows: if r<0.50 it signified low agreement; if r>0.50to0.69 indicated moderate agreement, r>0.70to0.89 indicated high agreement, and r>0.90 indicated very high agreement [61]. Bland-Altman plots were employed to assess the inherent variability of the data, where the scatter plot and narrow limit of agreement (LOA) signify a high degree of stability [62]. In addition, IBM® SPSS (version 21.0, IBM Corporation, Armonk, NY, USA) was utilized to conduct statistical analyses. This study was conducted at the main stadium of Chiang Mai University, Thailand. The time for 100-m sprint testing was around 9:00 a.m. to 12:00 a.m. on June 25, 2022.

## 4 Results

### 4.1 Participant characteristics

All participants were recruited from Chiang Mai University in Chiang Mai, Thailand. Five participants (3 males and 2 females). The mean age of the participants was 21.20 (1.10) years, the mean BMI was 21.78 (2.79) kg.m^−2^, the mean time of the first trial 100-m test was 16.682 (2.843) s, with an average speed 6.124 (0.953) m.s^−1^. And also, the mean time of the second trial test was 17.225 (2.213) s, with an average speed 5.887 (0.801) m.s^−1^. The participants successfully completed two tests without incident. The participants' demographic characteristics are shown in Table 1.

**Table 1:** Participants' demographic characteristics.

Participants	Characteristics								
	Gender	Age (years)	Height (cm)	Weight (kg)	BMI (kg.m^−2^)	100-m test			
						Time (sec)		Speed (m.s^−1^)	
						First Trial	Second Trial	First Trial	Second Trial
Subject 1	Male	20	168	68	24.1	14.211	13.955	7.037	7.166
Subject 2	Male	20	171	72	24.6	14.317	17.680	6.985	5.656
Subject 3	Male	22	180	73	22.5	16.724	16.876	5.980	5.925
Subject 4	Female	22	151	43	18.9	21.219	20.143	4.713	4.964
Subject 5	Female	22	160	48	18.8	16.937	17.470	5.904	5.724
Means (SD)		21.20 (1.10)	166.00 (11.02)	60.80 (14.20)	21.78 (2.79)	16.682 (2.843)	17.225 (2.213)	6.124 (0.953)	5.887 (0.801)

### 4.2 The 100-m performance of participants

**Fig. 3:**
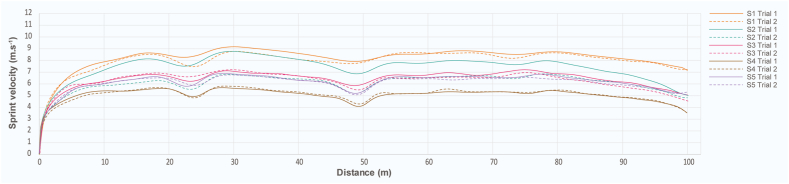
The sprint velocity curves of the participants (S1 to S5) with two trials of the 100-m speed test. Trial 1 shows the sprint velocity of each participant (solid line), and Trial 2 shows the sprint velocity of each participant (dash line).

The overall speed of all participants with two trials was collected by hand stopwatch. Fig. 3 illustrates the plotting of a sprinter's velocity-distance trajectory within 100 m for all trials from the computer vision-based system. At the same time, the collected data from web cameras was calculated by the computer vision-based system. Five participants performed two 100-m tests. The results indicated that the majority of the participants' speed slightly declined in the second trial.

### 4.3 Concurrent validity

The association between the proposed system and the video analysis software (Tracker) during the ten subtasks ranged from 0.961 to 1.000, indicating a strong relationship. The lowest correlation was observed in the meter 0 to 10 (V1) subtask (r = 0.961, p < 0.0001), while the highest agreement was found in the meter 10 to 20 (V2) subtask (r = 1.000, p < 0.0001). Table 2 presents the correlation between two systems and the mean speed value for each 100-m subtask.

**Table 2:** Correlation for the 100-m subtasks measures, as determined by the computer vision-based system, against those obtained from the video analysis software (Tracker).

100-m subtasks (m)	Speed (m.s^−1^)		r (95 % CI)	p-value^a^
	Tracker Mean (SD)	Computer vision-based Mean (SD)		
V1 (0–10)	4.819 (0.517)	4.736 (0.510)	0.961	0.0001
V2 (10–20)	6.678 (1.003)	6.695 (0.994)	1.000	0.0001
V3 (20–30)	7.209 (1.229)	6.758 (1.111)	0.996	0.0001
V4 (30–40)	7.049 (1.194)	6.991 (1.170)	0.999	0.0001
V5 (40–50)	6.483 (1.107)	6.253 (1.137)	0.995	0.0001
V6 (50–60)	6.660 (1.125)	6.554 (1.170)	0.998	0.0001
V7 (60–70)	6.842 (1.148)	6.850 (1.140)	0.999	0.0001
V8 (70–80)	6.857 (1.140)	6.861 (1.076)	0.992	0.0001
V9 (80–90)	6.533 (1.110)	6.543 (1.080)	0.998	0.0001
V10 (90–100)	5.688 (1.070)	5.716 (1.068)	0.999	0.0001

r = Correlation coefficients, CI = Confidence interval.

^a^ Significant difference at p < 0.0001.

**Fig. 4:**
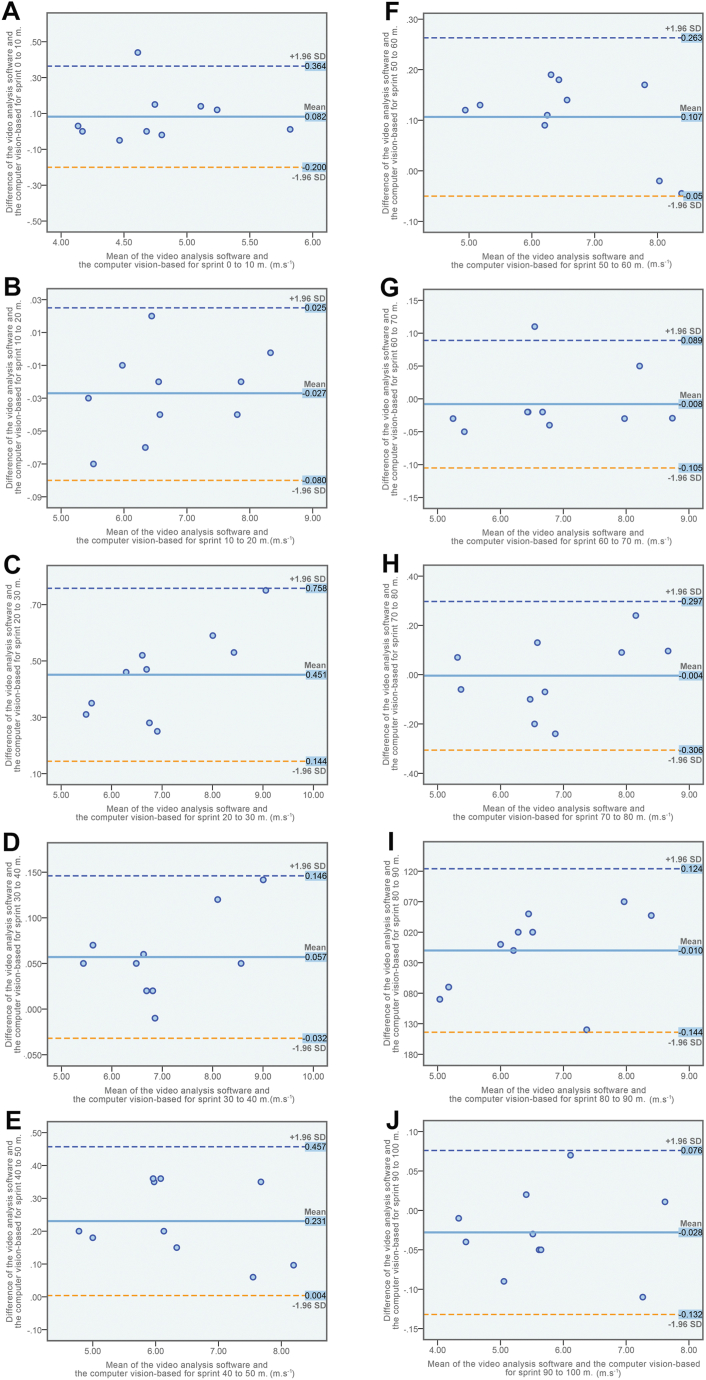
The Bland-Altman plots assessing concurrent validity in the context of the 100-m sprint test. The plots demonstrate the agreement between the computer vision-based system and the video analysis software (Tracker) for the measurement of the subtask speed of the 100-m test. (A) Sprint meter 0 to 10, (B) sprint meter 10 to 20, (C) sprint meter 20 to 30, (D) sprint meter 30 to 40, (E) sprint meter 40 to 50, (F) sprint meter 50 to 60, (G) sprint meter 60 to 70, (H) sprint meter 70 to 80, (I) sprint meter 80 to 90, and (J) sprint meter 90 to 100. The horizontal axis depicts the average values, while the vertical axis shows the average difference between the two systems for each subtask in the 100-m sprint test. Reference lines are utilized to illustrate the mean difference between the two systems (solid line) and the 95 % limit of agreement (LOA) for the mean difference (dashed line).

Additionally, Fig. 4 displays the Bland-Altman plots for the subtasks of the 100-m. For the majority of participants throughout the various subtasks of the 100-m event as reported in Fig. 4 (B–F, H, I, J), the mean differences observed between the two systems were found to be near zero, falling within the limits of agreement (95 % LOA). Additionally, the scatter plot distribution exhibited symmetry, indicating a high level of agreement between the systems. There were two outliers (10 %) across ten subtasks (meter 0 to 10, Fig. 4A) and (meter 60 to 70, Fig. 4G) in the 100-m speed test.

## 5 Discussion

The purpose of this study was to create a computer vision-based system capable of accurately measuring the instantaneous speed of a sprinter. Additionally, this study aims to evaluate the concurrent validity of the computer vision-based system that was built for the purpose of assessing the 100-m sprinting speeds of ten subtasks in athletes. We hypothesized that the computer vision-based system developed would have high concurrent validity in all subtask sprinting speeds of 0–100 m. In accordance with expectations, all of the subtasks' sprinting speeds presented a high degree of association between the two systems. Based on the available information, this research is the first investigation into the concurrent validity of using the video analysis software (Tracker) to accurately measure the sprinting speed of individual subtasks during the 100-m event among athletes.

The gold standard for quantifying 100-m subtask sprint speeds was Tracker. In the present investigation, the 100-m speed of each subtask was calculated using 90 to 150 instantaneous speed values from the computer vision-based system and Tracker. This study demonstrated that the correlation between the proposed system and Tracker was very high across all subtasks and trials of the 100-m sprint test. In addition, a visual examination of the Bland-Altman plots for concurrent validity revealed that the two systems were in good agreement. Our findings are consistent with prior research, which found that the computer vision-based approach outperformed video analysis tools in estimating instantaneous sprint speed in athletes [17,32,33,48]. Furthermore, the previous studies agreed that the video camera-based system had a higher degree of correlation than the force distribution measurement platform while evaluating 100-m speed in sprint athletes [31,32].

### 5.1 Limitations of the study

There were a few limitations to this study. The first relates to the background subtraction technique, which influences motion segmentation and feature extraction. Tracking the COM of a human body necessitates resolving significant environmental issues such as sky luminance, moving clouds, large drak objects, tree leaves, several moving small objects, and moving shadows, as well as the color of the clothing. Therefore, dark gray or black is the appropriate color for an athlete's suit, while other colors are prohibited. The second is capturing the volume of the system, which is dependent on camera placement and camera lens; for example, if the capture volume of the athlete racing around was 25 m, the camera must be located about 20–25 m away from the athlete's body. The third is the intersection between sources 1 and 4 from the actual location camera. As presented in Fig. 3, the velocity data are not consistent. It may pose a problem for the location detection algorithm. Finally, the small sample size may have an effect on statistical power; therefore, future studies with larger sample sizes would improve the power analysis.

### 5.2 Future works

Future studies need to develop an autonomous system for tracking location using deep learning techniques [48,49,[63], [64], [65], [66]] (object detection algorithms) to detect the speed and spatiotemporal of human movement (split times, split distance, step length, step length, and ground contact time) and machine learning techniques [67] to predict an individual athlete's training plan. Additionally, the system needs to be implemented with professional athletes, such as the Thai National Team.

## 6 Conclusion

In the present study, it was demonstrated that the computer vision-based system had high concurrent validity with the video analysis software (Tracker) for measuring 100-m subtask sprinting speeds. In addition, the Bland-Altman plots established that the computer vision-based system had good agreement with the Tracker and high temporal stability for measuring 100-m subtask sprinting speeds among athletes.

## Data availability statement

No data was used for the research described in the article.

## Lead contact website

Teerawat Kamnardsirihttps://www.camt.cmu.ac.th/index.php/en/about-us/list-of-camt-s-staff.html.

## CRediT authorship contribution statement

**Teerawat Kamnardsiri:** Writing – review & editing, Writing – original draft, Visualization, Validation, Software, Resources, Project administration, Methodology, Investigation, Funding acquisition, Formal analysis, Data curation, Conceptualization. **Sirinun Boripuntakul:** Writing – review & editing, Validation, Supervision, Project administration, Methodology, Investigation, Formal analysis, Data curation, Conceptualization. **Chinawat Kaiket:** Writing – review & editing, Visualization, Validation, Supervision, Project administration, Methodology, Investigation, Formal analysis, Data curation, Conceptualization.

## Declaration of competing interest

The authors declare the following financial interests/personal relationships which may be considered as potential competing interests:Chinawat Kaiket reports financial support was provided by 10.13039/501100002842Chiang Mai University.

## References

[1] Gurung R. (2023). Top 16 most popular sports in the world 2023. https://playersbio.com/most-popular-sports-in-the-world/.

[2] Babu Tendu C.A., Adrianna S. (2023). Top 20 most popular sports in the world revealed as of 2023. https://sportsbrief.com/other-sports/16715-revealed-top-popular-sports-world/.

[3] Verma M. (2023). Top 15 most popular sports in the world 2023. https://www.edudwar.com/most-popular-sports-in-the-world/.

[4] Staff T. (2022). https://www.totalsportal.com/list/most-popular-sports/#10_Athletics.

[5] Carr G.A. (1999).

[6] Jones R., Bezodis I., Thompson A. (2009). Coaching sprinting: expert coaches' perception of race phases and technical constructs. Int. J. Sports Sci. Coach..

[7] Ae M., Ito A., Suzuki M. (1992). The men's 100 metres. New Stud. Athl..

[8] Mackala K. (2007). Optimisation of performance through kinematic analysis of the different phases of the 100 metres. New Stud. Athl..

[9] ATHLETICS (2021).

[10] Gerhardt S. (1983).

[11] Mattes K., Wolff S., Alizadeh S., S (2021). Kinematic stride characteristics of maximal sprint running of elite sprinters–verification of the “swing-pull technique”. J. Hum. Kinet..

[12] Manzer S., Mattes K., Holländer K. (2016). Kinematic analysis of sprinting pickup acceleration versus maximum sprinting speed. Biol. Sport.

[13] Debaere S., Jonkers I., Delecluse C. (2013). The contribution of step characteristics to sprint running performance in high-level male and female athletes. J. Strength Condit Res..

[14] Mero A., Komi P., Gregor R. (1992). Biomechanics of sprint running: a review. Sports Med..

[15] Young M., Choice C. (2007). Maximal velocity sprint mechanics. Track Coach.

[16] Maćkała K., Fostiak M., Kowalski K. (2015). Selected determinants of acceleration in the 100m sprint. J. Hum. Kinet..

[17] Haugen T., Buchheit M. (2016). Sprint running performance monitoring: methodological and practical considerations. Sports Med..

[18] Quercetani R. (2006).

[19] Clark R.A., Pua Y.H., Bower K.J. (2019). Validity of a low-cost laser with freely available software for improving measurement of walking and running speed. J. Sci. Med. Sport.

[20] Arsac L.M., Locatelli E. (2002). Modeling the energetics of 100-m running by using speed curves of world champions. J. Appl. Physiol..

[21] Bezodis N.E., Salo A.I., Trewartha G. (2012). Measurement error in estimates of sprint velocity from a laser displacement measurement device. Int. J. Sports Med..

[22] Štuhec S., Planjšek P., Ptak M., Čoh M., Mackala K. (2022). Application of the laser linear distance-speed-acceleration measurement system and sport kinematic analysis software. Sensors.

[23] Morin J.B., Jeannin T., Chevallier B., Belli A. (2005). Spring-mass model characteristics during sprint running: correlation with performance and fatigue-induced changes. Int. J. Sports Med..

[24] Nagano A., Fujimoto M., Kudo S., Akaguma R. (2017). An image-processing based technique to obtain instantaneous horizontal walking and running speed. Gait Posture.

[25] Feser E.H., Neville J., Wells D., Diewald S., Kameda M., Bezodis N.E., Clark K., Nagahara R., Macadam P., Uthoff A.M., Tinwala F. (2023). Lower-limb wearable resistance overloads joint angular velocity during early acceleration sprint running. J. Sports Sci..

[26] Kamnardsiri T., Khuwuthyakorn P., Boripuntakul S. (2019, October). Proceedings of the 2019 4th International Conference on Biomedical Imaging.

[27] Kamnardsiri T., Thawinchai N., Parameyong A. (2023). Conventional video-based system for measuring the subtask speed of the Timed up and Go Test in older adults: validity and reliability study. PLoS One.

[28] Linke D., Link D., Lames M. (2020). Football-specific validity of TRACAB's optical video tracking systems. PLoS One.

[29] Stewart F., Turner P.N., Miller A.C., S (2014). Reliability, factorial validity, and interrelationships of five commonly used change of direction speed tests. Scand. J. Med. Sci. Sports.

[30] Haugen A., Tønnessen T.E., Seiler K., S (2012). The difference is in the start: impact of timing and start procedure on sprint running performance. J. Strength Condit Res..

[31] Chelly S., M, Fathloun M., Cherif N. (2009). Effects of a back squat training program on leg power, jump, and sprint performances in junior soccer players. J. Strength Condit Res..

[32] Harrison J., A, Jensen L., R, Donoghue O. (2005). A comparison of laser and video techniques for determining displacement and velocity during running. Meas. Phys. Educ. Exerc..

[33] Kamnardsiri T. (2018). Assessment the speed-up detection system for testing sprinters' performances using computer vision techniques. Eurasian. J. Anal. Chem..

[34] Dobre A.D., Gheorghe C. (2021). The optimization of the running technique using video analysis method. J. Phys.: Conf. Ser..

[35] Jha S., Seo C., Yang E., Joshi G.P. (2021). Real time object detection and trackingsystem for video surveillance system. Multimed. Tool. Appl..

[36] Balamuralidhar N., Tilon S., Nex F. (2021). MultEYE: monitoring system for real-time vehicle detection, tracking and speed estimation from UAV imagery on edge-computing platforms. Rem. Sens..

[37] Einfalt M., Lienhart R. (2020, June). Proceedings of the IEEE/CVF Conference on Computer Vision and Pattern Recognition Workshops.

[38] Freeman W., Talpey B.W., S, James P., L (2023). Common high-speed running thresholds likely do not correspond to high-speed running in field sports. J. Strength Condit Res..

[39] Pons E., García-Calvo T., Resta R., Blanco H., López del Campo R., Díaz García J., Pulido J.J. (2019). A comparison of a GPS device and a multi-camera video technology during official soccer matches: agreement between systems. PLoS One.

[40] Makar P., Silva F., A, Oliveira R. (2023). Assessing the agreement between a global navigation satellite system and an optical-tracking system for measuring total, high-speed running, and sprint distances in official soccer matches. Sci. Prog..

[41] Seidl T., Russomanno G., T, Stöckl M., Lames M. (2021). Assessment of sprint parameters in top speed interval in 100 m sprint—a pilot study under field conditions. Front. Sports Act. Living..

[42] Miranda-Oliveira P., Branco M., Fernandes O. (2023). Accuracy and interpretation of the acceleration from an inertial measurement unit when applied to the sprint performance of track and field athletes. Sensors.

[43] Feletti F., Bracco C., Takeko Molisso M., Bova L., Aliverti A. (2023). Analysis of fluency of movement in parkour using a video and inertial measurement unit technology. J. Hum. Kinet..

[44] de Ruiter C.J., Wilmes E., Brouwers S.A., Jagers E.C., van Dieën J.H. (2022). Concurrent validity of an easy-to-use inertial measurement unit-system to evaluate sagittal plane segment kinematics during overground sprinting at different speeds. Sports BioMech..

[45] Weinland D., Ronfard R., Boyer E. (2011). A survey of vision-based methods for action representation, segmentation and recognition. Comput. Vis. Image Underst..

[46] Eveland C., Konolige K., C, Bolles R. (1998, June). Proceedings of 1998 IEEE Computer Society Conference on Computer Vision and Pattern Recognition (Cat.

[47] Kamnardsiri T., Khuwuthyakorn P., Boripuntakul S., Janchai W. (2021). A knowledge-based Smart trainer system for transferring knowledge from coaches to long jump students. Front. Educ..

[48] Yagi K., Hasegawa K., Sugiura Y., Saito H. (2018, October). Proceedings of the 1st International Workshop on Multimedia Content Analysis in Sports.

[49] Stenum J., Rossi C., Roemmich R.T. (2021). Two-dimensional video-based analysis of human gait using pose estimation. PLoS Comput. Biol..

[50] Yang Y., Lee X., Enomoto Y. (2023). Implementing the tracking of 1500 m runners using Open CV. J. Phys. Educ. Sport..

[51] García-Pinillos F., Latorre-Román P.Á., Soto-Hermoso V.M., Párraga-Montilla J.A., Pantoja-Vallejo A., Ramírez-Campillo R., Roche-Seruendo L.E. (2019). Agreement between the spatiotemporal gait parameters from two different wearable devices and high-speed video analysis. PLoS One.

[52] Jafarzadeh P., Virjonen P., Nevalainen P., Farahnakian F., Heikkonen J. (2021, October). Proceedings of 2021 International Conference on Electrical, Computer, Communications and Mechatronics Engineering.

[53] Felipe J.L., Garcia-Unanue J., Viejo-Romero D., Navandar A., Sánchez-Sánchez J. (2019). Validation of a video-based performance analysis system (Mediacoach®) to analyze the physical demands during matches in LaLiga. Sensors.

[54] Hockicko P. (2014).

[55] Moeslund B., T, Moeslund B., T (2012).

[56] dos Santos Mesquita M., Halldórsdóttir S. (2005).

[57] Matsumura T., Tomoo K., Sugimoto T. (2023). Acute effect of caffeine supplementation on 100-m sprint running performance: a field test. Med. Sci. Sports Exerc..

[58] Healy R., Kenny I.C., Harrison A.J. (2022). Profiling elite male 100-m sprint performance: the role of maximum velocity and relative acceleration. J. Sport Health Sci..

[59] Brown D., Christian W., Hanson M., Tracker R. (2022). https://physlets.org/tracker/.

[60] Castaneda A. (2019). Rectilinear movement and functions through the analysis of videos with Tracker. Phys. Teach..

[61] Mukaka M.M. (2012). A guide to appropriate use of correlation coefficient in medical research. Malawi Med. J..

[62] Bland J.M., Altman D. (1986). Statistical methods for assessing agreement between two methods of clinical measurement. Lancet.

[63] Ren H. (2023). Sports video athlete detection based on deep learning. Neural Comput. Appl..

[64] Mhalla A., Chateau T., Amara N.E.B. (2019). Spatio-temporal object detection by deep learning: video-interlacing to improve multi-object tracking, Image Vis. Comput. Times.

[65] Einfalt M., Dampeyrou C., Zecha D. (2019, October). Proceedings Proceedings of the 2nd International Workshop on Multimedia Content Analysis in Sports.

[66] Mehta R., Amores J. (2018). Improving detection speed in video by exploiting frame correlation. Pattern Recogn. Lett..

[67] Richter C., O'Reilly M., Delahunt E. (2021). Machine learning in sports science: challenges and opportunities. Sports BioMech..

