# Supplementary material for: Computer vision-based instantaneous speed tracking system for measuring the subtask speed in the 100-meter sprinter: Development and concurrent validity study
Source: Heliyon. 2024 Jan 6;10(2):e24086. doi: 10.1016/j.heliyon.2024.e24086 (PMC11681224; doi:10.1016/j.heliyon.2024.e24086)
Supplement: Multimedia component 1 [file mmc1.pdf]

Supplemental Results

Sub-Trial	Tracker 0-10	Video 0-10	Tracker 10-20	Video 10-20	Tracker 20-30	Video 20-30	Tracker 30-40	Video 30-40	Tracker 40-50	Video 40-50	Tracker 50-60	Video 50-60	Tracker 60-70	Video 60-70	Tracker 70-80	Video 70-80	Tracker 80-90	Video 80-90	Tracker 90-10(	Video 90-100
S1T1	5.83	5.82	8.33	8.33	9.43	8.68	9.08	8.93	8.24	8.15	8.36	8.41	8.72	8.75	8.71	8.62	8.42	8.37	7.62	7.61
S1T2	5.18	5.04	7.78	7.82	8.30	7.71	8.16	8.04	7.58	7.52	8.02	8.04	8.24	8.19	8.27	8.03	8.00	7.93	7.21	7.32
S2T1	5.30	5.18	7.85	7.87	8.69	8.16	8.59	8.54	7.85	7.50	7.88	7.71	7.96	7.99	7.97	7.88	7.30	7.44	6.15	6.08
S2T2	4.83	4.39	5.97	5.98	6.52	6.06	6.51	6.46	6.15	5.80	6.25	6.16	6.60	6.49	6.65	6.52	6.29	6.27	5.42	5.40
S3T1	4.79	4.81	6.54	6.56	6.89	6.61	6.85	6.86	6.41	6.26	6.63	6.49	6.76	6.80	6.75	6.99	6.52	6.50	5.59	5.64
S3T2	4.68	4.68	6.55	6.59	7.03	6.78	6.82	6.80	6.23	6.03	6.40	6.21	6.43	6.45	6.44	6.64	6.00	6.00	5.01	5.10
S4T1	4.17	4.17	5.42	5.45	5.65	5.34	5.46	5.41	4.88	4.68	5.00	4.88	5.23	5.26	5.35	5.28	4.99	5.08	4.33	4.34
S4T2	4.15	4.12	5.48	5.55	5.78	5.43	5.66	5.59	5.09	4.91	5.24	5.11	5.40	5.45	5.34	5.40	5.14	5.21	4.43	4.47
S5T1	4.82	4.67	6.45	6.43	6.93	6.46	6.70	6.68	6.26	5.90	6.52	6.34	6.66	6.68	6.67	6.74	6.47	6.42	5.62	5.67
S5T2	4.44	4.49	6.31	6.37	6.87	6.35	6.66	6.60	6.14	5.78	6.30	6.19	6.42	6.44	6.42	6.52	6.20	6.21	5.50	5.53
